# The role of perceived social support for outcomes for the long-term health condition hypermobile Ehlers-Danlos syndrome

**DOI:** 10.1177/13591053251410690

**Published:** 2026-02-03

**Authors:** Kimberley C. Schenke, Joanna Foster, Danielle Stephens-Lewis

**Affiliations:** 1 University of Gloucestershire, Cheltenham, UK

**Keywords:** hypermobile Ehlers Danlos syndrome, companionship, dogs, canine companion, loneliness, health and wellbeing

## Abstract

Social connection is a key health determinant yet remains under-researched in long-term conditions marked by isolation such as hypermobile Ehlers-Danlos Syndrome (hEDS). Individuals with hEDS are especially vulnerable due to the condition’s complex and often misunderstood symptoms. This study presents the first high-powered quantitative investigation (*n* = 401) of perceived social support and companionship in hEDS. Using validated, theory-informed measures, we examined whether loneliness, support from family and friends, and dog guardianship predicted physical, mental, and social wellbeing outcomes. Loneliness was the most robust and consistent predictor, linked to poorer outcomes. Support from friends predicted some wellbeing indicators, whereas family support and dog guardianship had minimal predictive value. These findings have practical implications: interventions targeting loneliness and peer connection may be more effective than generalised or pet-based support. This research offers a novel contribution to understanding social determinants in chronic, stigmatised conditions, with relevance for intervention design and health policy.

## Introduction

Long-term health conditions are complex and multifaceted (Larsen, 2023), and often affect both physical and mental wellbeing (Harvey et al., 2020; Wilson and Stock, 2019). Whilst clinical management is crucial, there is increasing recognition for the role of perceived social support in outcomes. According to the stress-buffering model of social support (Cohen and Wills, 1985) supportive relationships can help protect individuals from the negative effects of stress and chronic illness. This has been supported by a wide body of research demonstrating that higher levels of perceived support are linked to improved outcomes across various health conditions. For example, research has shown improved outcomes associated with higher perceived support across a wide range of health conditions including depression (Cruwys et al., 2013; Iyer et al., 2009), acquired brain injury (Haslam et al., 2008; Kinsella et al., 2020; Walsh et al., 2015), and addiction (Best et al., 2016). In contrast, low levels of perceived support are linked to poorer outcomes, including worse physical (Cornwell and Waite, 2009; Galloway and Henry, 2014) and mental wellbeing (Cruwys et al., 2014; Wickramaratne et al., 2022). Moreover, perceived social isolation or loneliness present an increased risk of depression (Cacioppo et al., 2010; Taylor et al., 2018), cognitive decline (Cacioppo and Hawkley, 2009), heart disease (Valtorta et al., 2016), diminished physical function (Shankar et al., 2017), and early mortality (Holt-Lunstad et al., 2015).

Although much of this work focuses on human relationships, research is increasingly suggesting that companion animals may also provide meaningful support and reduce social isolation (Brooks et al., 2013; Oliva and Johnston, 2021; Stephens-Lewis and Schenke, 2023). This may be especially valuable for individuals with lesser-known or ‘invisible’ health conditions, where social misunderstanding, stigma, and inadequate treatment contribute to psychological burden (Anderson and Lane, 2022; Langhinrichsen-Rohling et al., 2021).

One such condition is Ehlers Danlos Syndrome (EDS) - a group of 13 confirmed and agreed upon heterogenous conditions affecting the connective tissue (Malfait et al., 2020). Prevalence estimates vary widely, from 1 in 227 in a Northumberland study (Saravanan et al., 2024) to 1 in 500 in Wales (Demmler et al., 2019), reflecting inconsistencies in diagnosis and limited understanding of the condition’s true frequency (Kulas Søborg et al., 2017; Sinibaldi et al., 2015). The hypermobile sub-type (hEDS) accounts for approximately 80% of cases (Tinkle et al., 2017). Typical physical symptomology includes chronic pain, joint hypermobility, fragile and hyperextensible skin, urogynaecological, cardiovascular and gastrointestinal complaints (Clark et al., 2023; Doolan et al., 2023). However, in addition to physical symptoms, individuals with hEDS face elevated rates of anxiety, depression, and suicidal ideation (Baeza-Velasco et al., 2019; Bulbena et al., 2017; Pasquini et al., 2014). These difficulties are often compounded by inadequate treatment options and profound disruptions to work, social, and family life (Palomo-Toucedo et al., 2020; Rombaut et al., 2011), which can significantly impact overall mental wellbeing (Orenius et al., 2022). Subsequently, the complex interplay between condition complications and the lack of effective treatment (and the indirect negative impact on social functioning) can result in those diagnosed with the condition becoming withdrawn and isolated (De Baets et al., 2022).

As such, research into psychosocial factors that could buffer the impact of long-term health conditions such as hEDS is urgently needed. Social support is one such factor, yet the nature and effectiveness of support from different sources (e.g., family, friends, animals) remains underexplored. Given their role as ‘best friend’ to their human guardian, there has been an increasing focus on the impact of canine companionship on human health and wellbeing (Barcelos et al., 2020; Christian et al., 2018; Merkouri et al., 2022; Morales-Jinez et al., 2018). While dogs are increasingly acknowledged as sources of comfort and non-judgemental companionship (Carr et al., 2018; Stephens-Lewis and Schenke, 2023), their specific contribution within hEDS has not been empirically tested. Previous findings in other chronic pain conditions suggest that dog guardianship may be associated with improved mood, reduced fatigue, better sleep, and greater pain acceptance (Baiardini et al., 2022; Brown et al., 2018; Carr et al., 2018; Silva et al., 2021). Lower anxiety and depression rates, and an enhanced quality of life have also been documented within other conditions including fibromyalgia (Silva et al., 2021) and arthritis (Thiele et al., 2023).

However, many of these studies lack rigorous theoretical grounding and do not disaggregate the effects of different sources of support. Moreover, not all individuals benefit from dog companionship, and outcomes vary depending on context and condition (Wells, 2009). To advance theoretical clarity and empirical evidence, the current study applies the stress-buffering model to evaluate the associations between three distinct sources of perceived support - family, friends, and companion dogs - and a range of health and wellbeing outcomes in individuals with hEDS.

Such research is particularly pertinent given that the social isolation often felt by those with hEDS (De Baets et al., 2022; Palomo-Toucedo et al., 2020) may mean that canine companionship could be particularly beneficial to this population. The potential benefits arising from the increased physical activity and stress reduction associated with dog guardianship (Christian et al., 2018; Janssens et al., 2020; Westgarth et al., 2017; Wheeler and Faulkner, 2015), combined with the social facilitation role dogs can play (Bould et al., 2018; Wood et al., 2015), may provide an alternative avenue to support the long-term management of hEDS (Carr et al., 2018; Stephens-Lewis and Schenke, 2023).

Loneliness was also included in the current study because of its well-documented associations with mental and physical decline (Cacioppo et al., 2010; Holt-Lunstad et al., 2015), and its prevalence among people with hEDS (De Baets et al., 2022). Therefore, informed by prior research and theory, it was hypothesised that higher perceived social support from family and friends, presence of a canine companion, and lower levels of loneliness would be associated with better outcomes across measures of wellbeing, physical health, general health, pain, fatigue, depression, and anxiety.

## Method

The current study forms part of a broader investigation into health and wellbeing outcomes among individuals diagnosed with hEDS, with a specific focus here on the roles of perceived social support (from family, friends, and canine companions) and loneliness. Ethical approval was obtained from the University of Gloucestershire ethics committee.

A comprehensive overview of the methodology for the wider study (alongside the complete dataset), which investigated health and wellbeing in individuals with a hEDS diagnosis living with or without a canine companion is available in Foster et al. (2025). The research team consisted of a doctoral researcher with expertise in hEDS, a researcher with expertise in human-dog interaction and health research, and a researcher with both expertise and lived experience of hEDS.

### Participants

For the current hypotheses, prospective power analyses assuming a medium effect size with an alpha level of 0.05 suggested a minimum of 67 participants for 0.80 power (G power; Faul et al., 2007). Four hundred and sixteen participants were recruited through social media sites, the EDS society website and the RIC:HER Alliance (consisting of health professionals and experts by experience) based on having a diagnosis of hEDS and being at least 18 years of age. However, one participant was excluded for not having a hEDS diagnosis, and 14 participants were excluded for living with a registered assistance dog, which could represent a confounding source of support. Therefore, the final sample consisted of 401 participants (*Mean* age = 38.4, SD = 12.0; 375 female, 10 male, 12 non-binary, 2 transgender, 2 ‘other’ – see Supplemental Materials for further information on the participant characteristics) most of whom resided in England (195) or the UK more widely (104).

### Design, materials and procedure

As aforementioned, this research is situated within a wider study, which investigated the influence of canine companionship on health and wellbeing outcomes of people living with hEDS. A cross-sectional design was employed using an online survey hosted on JISC OnlineSurveys. Participants provided informed consent and completed demographic questions, items about their health condition, dog guardianship, and a battery of validated psychological measures before being thanked and debriefed:

- Quality of Life: RAND 36-Item Health Survey (SF-36; Hays et al., 1993). Higher scores reflect better health states. The role limitations due to physical health subscale was excluded due to technical issues in recording responses. Reliability estimates were high, apart from energy/fatigue and social functioning subscales, (see Foster et al., 2025) likely due to ceiling effects, a known limitation in chronic illness populations (Amtmann et al., 2012; Murdock et al., 2017).
- Subjective Wellbeing: Warwick-Edinburgh Mental Wellbeing Scale (Tennant et al., 2007). Higher scores represent greater wellbeing. Cronbach’s alpha = 0.90.
- Physical Health: Physical Health Questionnaire (PHQ; Schat et al., 2005). Higher scores represent poorer physical health. Cronbach’s alpha = 0.81.
- Generalised Anxiety: Generalised Anxiety Disorder-7 (GAD-7; Spitzer et al., 2006). Higher scores indicate greater anxiety. Cronbach’s alpha = 0.92.
- Depression: Patient Health Questionnaire-9 (PHQ-9; Kroenke et al., 2001). Higher scores indicate greater depressive symptoms.
- Chronic Pain Acceptance: Chronic Pain Acceptance Questionnaire-8 (CPAQ-8; Fish et al., 2010). Higher scores reflect better acceptance of chronic pain. Cronbach’s alpha = 0.78.
- Loneliness: UCLA-3 Loneliness Scale (Hughes et al., 2004). Higher scores indicate greater loneliness. Cronbach’s alpha = 0.87.
- Perceived Social Support: Perceived Social Support-Friends (PSS-Fr) and Perceived Social Support-Family (PSS-Fa) scales (Procidano and Heller, 1983) were used to assess the perceived availability and adequacy of emotional and instrumental support from friends and family members. Higher scores indicate a greater sense of being supported and understood by these groups. Cronbach’s alphas were 0.91 (friends) and 0.93 (family).
- Dog Guardianship: Participants were asked whether they currently lived with a pet dog (yes, no). Those who did were also asked about their attachment to the dog and the behaviour of their dog (see Schenke et al., 2025).

For this paper we focus on the sections pertaining to companionship, including measures of human companionship and the presence of canine companions. This subset of the study employed a cross-sectional design with four predictor variables; canine companionship (pet dog; 74.1% of participants, no pet dog; 25.9% of participants), loneliness, and perceived social support from family and friends (separately). Each of the following criterion variables were analysed via separate multiple regressions; wellbeing, physical health, general health, health-related quality of life, health compared to a year ago, pain, depression and anxiety.

## Results

For further context on the hEDS characteristics of the participants, their co-occurring conditions and treatments reported please see Foster et al. (2025) and the Supplemental Materials.

Most participants felt they had companionship from multiple sources (see Table 1). The most frequently reported was from their canine companion, closely followed by their family and friends. Many participants (317) reported living with a spouse or partner or living with other adults (these were listed as family members for 98 participants). One hundred and twenty-five participants lived with their child(ren), whilst 48 did not. Seventy-two participants lived alone, two participants lived in residential accommodation and two lived in temporary accommodation. Six participants did not disclose their living situation. Additionally, a large proportion (161) reported being married or in a civil partnership, 103 reported being in a relationship, while 81 were not currently in a relationship, and 51 reported never having been married or in a civil partnership. Five preferred not to disclose their relationship status and one participant did not provide a response.

**Table 1.** The frequency of reporting of sources of companionship.

Source of companionship	Frequency
Your pet(s)	285 (71%)
Family	284 (70.8%)
Friends (face-to-face)	212 (52.9%)
Friends (online)	192 (47.9%)
Acquaintances	70 (17.5%)
Other	41 (10.2%)
Support group(s)	40 (10.0%)

To address missing data the mean scores for each participant for each instrument were imputed following reverse coding (aside from the SF-36, which had specific instructions to ignore missing data). Where there were no responses provided for a questionnaire, the participant was removed from the relevant analyses.

A series of multiple regression analyses using the Enter method were conducted to examine whether perceived social support (from friends and family separately), loneliness, and dog guardianship predicted a range of health and wellbeing outcomes supported by robust regression analyses of iterated re-weighted least squares using Huber weights and bisquare weighting using the ‘Mass’ (Venables and Ripley, 2002) and ‘Foreign’ (R Core Team, 2023) r packages (see Supplemental Materials for the r script). The same analyses were also conducted based on the non-imputed data, but none of these additional analyses changed the interpretation so they are not reported. All reported *p* values are two-tailed.

Across models, loneliness consistently emerged as the strongest and most reliable predictor, significantly associated with poorer outcomes in wellbeing, anxiety, pain acceptance, general health, social functioning, physical functioning, and fatigue (for all *p* ≤ 0.002). Perceived support from friends significantly predicted outcomes such as wellbeing (*p* < 0.001), anxiety (*p* < 0.001), general health (*p* = 0.046), and pain acceptance (though only in the CPAQ-8- *p* = 0.010 -, not in the SF-36), but to a lesser extent than loneliness. Support from family was only significantly associated with wellbeing (*p* < 0.001). Dog guardianship was only significantly associated with role limitations due to emotional health (*p* = 0.027), but the effect size was small. No predictor variables were associated with change in health over the past year or with overall health-related quality of life. Full regression outputs, including coefficients and diagnostics, are reported in Supplemental Materials.

## Discussion

The current research is the first high-powered investigation, to our knowledge, into perceived social support from family and friends, canine companionship and feelings of loneliness and health and wellbeing in individuals with hEDS - a population at elevated risk for physical and emotional distress. It employed validated measures, robust analyses and a theoretically grounded model to distinguish between different sources of support, and their relative associations with a wide range of outcomes, including physical and social functioning, pain, fatigue, and psychological wellbeing.

As hypothesised, the overall regression model was statistically significant for all health and wellbeing outcomes. Loneliness emerged as the most robust predictor, significantly associated with poorer outcomes on all variables except perceived health change and physical health. The strength and independence of this effect, evidenced by large semi-partial correlations, confirms loneliness as a core risk factor for individuals living with hEDS - supporting findings in the broader literature (e.g., Park et al., 2020). This highlights the urgency of developing interventions specifically targeting loneliness in this group.

Perceived support from friends was also a consistent and meaningful predictor, significantly associated with wellbeing, anxiety, general health, and functioning outcomes. Its contribution, although smaller than that of loneliness, suggests that friendship-based support may buffer the psychological burden of hEDS, possibly because such relationships are more voluntary, reciprocal, or emotionally validating than familial ties (Zhang and Dong, 2022). This is important because hEDS is often associated with reduced social networks and difficulty maintaining peer relationships due to its symptomology and misunderstandings (De Baets et al., 2022; Palomo-Toucedo et al., 2020). However, an important direction for future research, which was beyond the scope of the current study, is to assess the size, quality, and stability of friendships in those with long term health conditions such as hEDS.

In contrast, support from family was only a weak predictor, reaching significance for wellbeing and social functioning but with low unique variance. These findings suggest that not all support is experienced equally and may reflect limitations or ambivalence of family-based support, particularly in stigmatised or misunderstood conditions (Bennett et al., 2021). These findings replicate broader evidence that suggests the support offered by friends has a greater impact on reducing loneliness than the support of family (Zhang and Dong, 2022), highlighting the nuanced role of family relationships. It may be that emotional availability, responsiveness, or perceived validation are more critical than mere presence or support frequency. Therefore, future research should explore the relational dynamics and emotional salience of family support in the context of chronic conditions.

This is particularly important for those with ‘invisible’ or lesser understood conditions like hEDS, who are often accused of hypochondria by family members, with suggestions that symptoms are all in the mind (Bennett et al., 2021; Palomo-Toucedo et al., 2020). The sporadic nature and invisibility of symptoms can make it difficult to share experiences with, and gain acceptance, even from close family members, and many struggle both to ask for, and accept, help (Bennett et al., 2021). Moreover, hEDS symptoms may impact on physical intimacy (Johansen et al., 2021; Palomo-Toucedo et al., 2020). As such their experiences with hEDS may have contributed to breakdowns in family relationships. Whilst there is no research evidence to support this to date, the many anecdotal reports suggest this is a key avenue for further research. Moreover, it is important for future research to explore those situations (such as where medical stigma or disbelief are common) where social interactions may not be experienced in a positive or accessible manner.

Dog guardianship was significantly associated with only one outcome: role limitations due to emotional health. That is, participants reported fewer limitations relating to how emotional health issues impacted their ability to perform daily activities and fulfil roles within work, family and social interactions. While this partially aligns with hypotheses and previous studies suggesting that dogs may provide emotional buffering and support without ‘judgement’ (Carr et al., 2018), the small effect sizes and null findings across other outcomes challenge assumptions about the generalisability of pet-based support. These findings also align with several studies highlighting the contradictory findings of the impact of canine companionship on human health and wellbeing (Wells, 2009).

Several possible explanations merit further exploration. First, the human-dog relationship may not be universally positive. Some participants reported dogs with behavioural issues, which may reduce benefits or introduce stress (Bradley and Bennett, 2015; Buller and Ballantyne, 2020). Whilst beyond the scope of the current article, we have explored this possibility alongside others (including whether the participant was having a flare up in their hEDS at the time of the questionnaire and how much time they typically spend with their dog) within the current data in Schenke et al. (2025). We found that those reporting more dog behavioural issues were associated with worse outcomes. Additionally, 107 participants reported that their dog had its own health conditions, which may have particularly influenced the mental health of participants if they were worried or concerned about their pet. This may be particularly problematic if hEDS symptoms impaired their ability to support their pet, or if their pet’s condition negatively impacted their hEDS.

Second, not all participants were the primary caregiver (76 reported being their secondary caregiver, 29 reported only cohabiting with their dog, 2 were not involved in their care at all and 1 did not respond as to their relationship) and some who were the primary caregiver may have acquired their dog as a coping strategy for isolation, rather than a source of ongoing support. Thus, it is important to consider the relationship between the human and dog because wellbeing is typically higher in those that spend more time with their pets (Barklam and Felisberti, 2023). There is also some evidence that other household relationships could negatively influence the effect of pet guardianship (Himsworth and Rock, 2013). Therefore, both avenues are important for future research.

Third, evidence suggests that physical health benefits are felt more by those who otherwise live without adequate human support (Pruchno et al., 2018). However, in contrast, low levels of perceived social support have been associated with higher levels of depression and anxiety among dog guardians both in the general population (Duvall Antonacopoulos and Pychyl, 2010) and those living with chronic conditions (Silva et al., 2021). Although moderation was not formally tested here, preliminary exploration suggests a stronger anxiety-buffering role of dog guardianship for individuals with higher perceived support from friends. This raises critical theoretical questions around whether companion animal support amplifies, supplements, or substitutes human relationships, and how individual beliefs, expectations, or attachment styles shape the outcomes of pet guardianship. These are essential avenues to theoretically advance the field. Thus, it is important to consider the complexity of human-animal interaction research (Utz, 2014) and the need for person-centred, context-sensitive frameworks.

The lack of relationship between dog guardianship and human health and wellbeing may also be affected by the often debilitating nature of hEDS. Indeed, evidence suggests that the positive benefits of canine companionship are not consistent across all populations (Carr et al., 2020). Thus, further research is required to examine the mechanisms involved between the human-animal relationship and condition-specific physiological and psychological health outcomes. For example, it may be that participants with dogs felt guilty that they were not able to give their dog the life they wanted to (i.e., they may not have been able to go for as many walks with their dog, or engage in as many shared activities as they would like) as reported among other populations (Merkouri et al., 2022; Westgarth et al., 2019). This would support previous research highlighting the importance of guardian expectations within canine companionship (Stephens-Lewis and Schenke, 2023).

As such it may be that dog guardianship has a more indirect effect on health and wellbeing by reducing loneliness (Oliva and Johnston, 2021) and isolation (Liu et al., 2019). Indeed, there is some evidence from investigations into the effects of the lockdowns resulting from the SARS-CoV-2 (COVID-19) global pandemic that the effects of social isolation and physical confinement could be mitigated by pet guardianship (Chopik et al., 2025). Another indirect effect could be on supporting other human relationships. For example, there is evidence that pets can increase incidental social interaction and the forming of new friendships (Wood et al., 2015).

Methodological limitations include the cross-sectional and self-report nature of the current study, which precludes causal inference and may be influenced by current mood or symptom fluctuations. Future work should incorporate longitudinal and mixed method designs to capture dynamic changes in support, loneliness, and wellbeing. Indeed, qualitative or mixed methods research would enable a deeper exploration of the way in which participants actually *experience* social connection and support to build a more rounded knowledge base on the topic. The prominence of themes such as loneliness, social misunderstanding, and experiences of not being believed or understood suggests that future research in this area should adopt more co-produced, participatory approaches. Such approaches would help ensure that the voices of people with hEDS are embedded within the research process itself, promoting what Fricker (2007) refers to as epistemic justice - the fair inclusion and valuing of lived experience as a legitimate and credible form of knowledge. Whilst the current research included a researcher with lived experience of hEDS, by engaging with further individuals with hEDS as collaborators rather than solely as participants, future work can better address the social and epistemic inequalities that contribute to feelings of isolation and invalidation.

Whilst the current research utilised existing, standardised measures to improve the overall rigour of the study, it is important to be aware that those around perceived support were based on quite general support (such as feeling like they had someone to talk to). This was an important first step to take within a hEDS sample, but further research needs to consider the nuanced support required by those living with complex long-term health conditions such as hEDS, which likely goes beyond the more general aspects of social support typically needed by the wider population. Although this study operationalised social support using the Perceived Social Support – Friends and Family Scales (Procidano and Heller, 1983), which conceptualise support in terms of the perceived availability and adequacy of emotional and instrumental help from close social networks, the findings indicate that social support may not function as a uniform construct across individuals with hEDS. Standardised measures such as these are valuable for quantifying perceived support but may not fully capture the diverse ways in which individuals experience or access support. Intersecting factors such as gender, age, disability, neurodivergence, and co-occurring conditions (e.g., chronic pain, fatigue, or mental health differences) likely influence both the accessibility and meaning of support. Future research would, therefore, benefit from adopting a more intersectional and context-sensitive approach - potentially integrating quantitative and participatory methods - to better understand how social and structural factors shape the experience of connection and loneliness in hEDS.

Our findings suggest that loneliness is a potent and modifiable risk factor, and that fostering meaningful friendships may offer protective effects, whereas passive forms of support (such as pet guardianship or general family presence) may be insufficient. For patients with hEDS, targeted efforts to promote social integration and provide tailored mental health support are likely to be more beneficial than companionship alone. These insights are also likely to apply to other under-recognised or stigmatised long-term conditions, where social disconnection is often overlooked.

Given the central role of loneliness, these findings offer actionable guidance for healthcare providers, commissioners, and patient organisations. Health services and advocacy groups should prioritise interventions that actively build social connection, such as peer mentoring schemes, virtual support groups, or friend-focused psychoeducation. Primary care providers should also be trained to identify and respond to loneliness and social disconnection as part of routine care for hEDS (and other long term health conditions), especially as these factors may exacerbate symptoms such as pain and fatigue.

While pets are often recommended as a source of comfort, clinicians and therapists should carefully weigh the physical and emotional demands of pet guardianship against the individual’s capacity, caregiving responsibilities, and broader social context. The development of screening tools or clinical guidelines could support more informed, person-centred decisions and help identify potential stressors for vulnerable individuals.

In parallel, public and family-facing education campaigns could play a crucial role in improving awareness of hEDS (and other ‘invisible’ conditions) and reducing stigma. By fostering greater understanding of the condition - particularly its invisible symptoms - such efforts could enhance the quality of social support from family members and reduce the likelihood of harmful misunderstandings or dismissive attitudes.

It is also important to consider that neurodivergence (highly represented in hEDS) may contribute to the ways in which loneliness and social connection are experienced by individuals with hEDS. For example, differences in social communication, sensory processing, and cognitive style may shape how social interaction and belonging are navigated and may partly account for the heterogeneity observed in the current findings. Brief feelings of social disconnection, for instance, might reflect not only limited social opportunity but also sensory overload, social fatigue, or mis-attuned communication. Future research should explore these links more directly, integrating perspectives from neurodiversity research to capture the complexity of social experience in this population.

More broadly, these findings highlight the importance of intersectionality in understanding loneliness and social support within the hEDS community. Experiences of connection are likely to be shaped not only by neurodivergence but also by overlapping aspects of identity and circumstance, including gender, ethnicity, socioeconomic status, and access to healthcare. Considering these intersections would promote a more inclusive and ecologically valid understanding of social experience in hEDS and support the development of more tailored interventions.

## Conclusion

The current research was the first, to our knowledge, to provide a theoretically grounded and high-powered examination of loneliness, perceived support, and canine companionship in individuals with hEDS. The findings strongly reinforce the central role of loneliness and, to a lesser extent, support from friends as predictors of health and wellbeing. In contrast, support from family and dog guardianship showed limited associations with outcomes. These results provide novel insights into the differential association of various sources of support with health and wellbeing, with practical implications for interventions and healthcare policy.

As loneliness emerged as a more powerful determinant of wellbeing than either human or animal companionship alone, targeted, theory-informed social connection interventions may offer the most immediate and scalable route to improving quality of life in hEDS and other under-supported chronic health conditions. Indeed, the findings highlight the importance of targeted interventions that prioritise social connectedness and emotional validation over general assumptions of support or pet guardianship.

By directly contributing to the evidence base for integrated care strategies and social prescribing models, this study supports a more nuanced and person-centred approach to managing complex chronic conditions. Future research should build on these findings through longitudinal and qualitative methods, engaging deeply with the lived experiences of individuals with hEDS to further refine theory and inform practice.

## Supplemental Material

sj-docx-1-hpq-10.1177_13591053251410690 – Supplemental material for The role of perceived social support for outcomes for the long-term health condition hypermobile Ehlers-Danlos syndrome Supplemental material, sj-docx-1-hpq-10.1177_13591053251410690 for The role of perceived social support for outcomes for the long-term health condition hypermobile Ehlers-Danlos syndrome by Kimberley C. Schenke, Joanna Foster and Danielle Stephens-Lewis in Journal of Health Psychology

## Data Availability

The raw data is available via the Open Science Framework: https://osf.io/ezmkv/files/osfstorage/66e0484143468d4234a3c853
