# Supplementary material for: The role of perceived social support for outcomes for the long-term health condition hypermobile Ehlers-Danlos syndrome
Source: J Health Psychol. 2026 Feb 3;31(12):4858–71. doi: 10.1177/13591053251410690 (PMC13612873; doi:10.1177/13591053251410690)
Supplement: Supplementary file 1 [file sj-docx-1-hpq-10.1177_13591053251410690.docx]

**Supplementary materials**

*Table 1. Participant characteristics*

| Participant Characteristics | With Dogs  (*n* = 297) | Without Dogs  (*n* = 104) |
| --- | --- | --- |
| *Ethnicity* |  |  |
| *British/English/Scottish/Welsh/Northern Irish* | 253 (85.2%) | 91 (87.5%) |
| *Irish* | 4 (1.3%) | 0 (0.0%) |
| *Other white background* | 25 (8.4%) | 7 (6.7%) |
| *White/Black Caribbean* | 1 (0.3%) | 0 (0.0%) |
| *White/Black African* | 1 (0.3%) | 0 (0.0%) |
| *White/Asian* | 1 (0.3%) | 0 (0.0%) |
| *Any other mixed background* | 7 (2.4%) | 1 (1.0%) |
| *Indian* | 1 (0.3%) | 0 (0.0%) |
| *Chinese* | 0 (0.0%) | 1 (1.0%) |
| *Any other ethnic group* | 1 (0.3%) | 2 (1.9%) |
| *Prefer not to say* | 3 (1.0%) | 0 (0.0%) |
| *Age Group* |  |  |
| *18–24 years* | 44 (14.8%) | 16 (15.4%) |
| *25–34 years* | 79 (26.6%) | 29 (27.9%) |
| *35–44 years* | 79 (26.6%) | 26 (25.0%) |
| *45–55 years* | 69 (23.2%) | 17 (16.3%) |
| *55–64 years* | 25 (8.4%) | 10 (9.6%) |
| *65+ years* | 1 (0.3%) | 6 (5.8%) |
| *Gender* |  |  |
| *Female* | 283 (95.3%) | 92 (88.5%) |
| *Male* | 7 (2.4%) | 3 (2.9%) |
| *Transgender* | 1 (0.3%) | 1 (1.0%) |
| *Non-binary* | 6 (2.0%) | 6 (5.8%) |
| *Other* | 0 (0.0%) | 2 (1.9%) |
| *Prefer not to say* | 0 (0.0%) | 0 (0.0%) |
| *Living Status* |  |  |
| *Living with spouse/partner* | 167 (56.2%) | 52 (50.0%) |
| *Living alone* | 41 (13.8%) | 33 (31.7%) |
| *Living with other adults* | 81 (27.3%) | 17 (16.3%) |
| *Living in temporary/residential accommodation* | 2 (0.7%) | 2 (1.9%) |
| *Prefer not to say* | 6 (2.0%) | 0 (0.0%) |
| *Employment Status* |  |  |
| *Full-time employment* | 84 (28.3%) | 28 (26.9%) |
| *Part-time employment* | 53 (17.8%) | 18 (17.3%) |
| *Student* | 28 (9.4%) | 10 (9.6%) |
| *Retired* | 11 (3.7%) | 7 (6.7%) |
| *Homemaker* | 14 (4.7%) | 3 (2.9%) |
| *Not working, long-term sick leave* | 67 (22.6%) | 21 (20.2%) |
| *Not working, seeking employment* | 3 (1.0%) | 1 (1.0%) |
| *Not working, other* | 35 (11.8%) | 15 (14.4%) |
| *Prefer not to say* | 2 (0.7%) | 1 (1.0%) |
| *Relationship Status* |  |  |
| *In a relationship* | 68 (22.9%) | 35 (33.7%) |
| *Married/civil partnership* | 132 (44.4%) | 29 (27.9%) |
| *Never married/civil partnership* | 35 (11.8%) | 16 (15.4%) |
| *Not in a relationship* | 57 (19.2%) | 24 (23.1%) |
| *Prefer not to say* | 5 (1.7%) | 0 (0.0%) |
| *Children (age)* |  |  |
| *Yes (<18)* | 59 (19.9%) | 19 (18.3%) |
| *Yes (>18)* | 45 (15.2%) | 18 (17.3%) |
| *Yes (both under and over 18)* | 28 (9.4%) | 3 (2.9%) |
| *No* | 163 (54.9%) | 63 (60.6%) |
| *Prefer not to say* | 2 (0.7%) | 1 (1.0%) |
| *Ethnicity* |  |  |
| *British/English/Scottish/Welsh/Northern Irish* | 253 (85.2%) | 91 (87.5%) |
| *Irish* | 4 (1.3%) | 0 (0.0%) |
| *Other white background* | 25 (8.4%) | 7 (6.7%) |
| *White/Black Caribbean* | 1 (0.3%) | 0 (0.0%) |
| *White/Black African* | 1 (0.3%) | 0 (0.0%) |
|  |  |  |

Table 2 details who diagnosed the hEDS, with the data split based on whether the participant had a canine companion or not. Note that there were more participants with than without a canine companion. However, in terms of diagnostician, a very similar pattern is seen across the two groups in that the majority were diagnosed by a rheumatologist, geneticist or an unspecified consultant. This is likely to include both participants who were diagnosed by two specialists simultaneously and those who have received two diagnoses, such as pre and post 2017.

*Table 2. Responsibility for diagnosis*

| Role/title | With canine companions  n (%) | Without canine companions  n (%) |
| --- | --- | --- |
| *Cardiologist* | 3 (1) | 1 (1) |
| *Dermatologist* | 1 (0.3) | 0 |
| *Gastroenterologist* | 2 (0.7) | 0 |
| *GP/ Primary care physician / nurse* | 16 (5.4) | 4 (3.8) |
| *Geneticist* | 43 (14.5) | 8 (7.7) |
| *Neurologist* | 1 (0.3) | 0 |
| *Orthopaedic specialist/ clinic* | 3 (1) | 1 (1) |
| *Osteopath* | 0 | 1 (1) |
| *Paediatrician* | 0 | 1 (1) |
| *Physiotherapist* | 5 (1.7) | 1 (1) |
| *Rheumatologist* | 178 (59.9) | 71 (68.3) |
| *Consultant unspecified* | 29 (9.8) | 10 (9.6) |
| *Role not specified* | 4 (1.3) | 2 (1.9) |
| *Combined diagnosis^1^* | 9 (3) | 3 (2.9) |
| *Unsure* | 3 (1) | 1 (1) |
| Total | **297** | **104** |

Importantly, most of our sample reported using therapeutic treatments and a pharmaceutical intervention (see Table 3), again with similar patterns for those with and without canine companions.

*Table 3. Medical Interventions*

| Intervention | With canine companions  n (%) | Without canine companions  n (%) |
| --- | --- | --- |
| *Therapeutic* |  |  |
| *One or more physio /massage therapies^1^* | 144 (48.5) | 44 (42) |
| *One or more psychological therapies^2^* | 4 (1.3) | 3 (2.9) |
| *Pain clinic* | 20 (6.7) | 3 (2.9) |
| *Splits/supports^3^* | 94 (31.6) | 28 (26.9) |
| *Mobility aids* | 18 (6.1) | 8 (7.7) |
| *Total using therapeutic treatments* | **179 (60.3)** | **51 (49)** |
|  |  |  |
| *Pharmaceutical* |  |  |
| *Pain* | 80 (26.9) | 20 (19.2) |
| *Anti-depressant/ anxiety* | 14 (4.7) | 1 (1) |
| *Allergy* | 4 (1.3) | 1 (1) |
| *Gastric* | 15 (5.1) | 4 (3.8) |
| *Co-morbidity* | 12 (4) | 2 (1.9) |
| *Steroids* | 5 (1.7) | 1 (1) |
| *Other / unspecified^5^* | 79 (26.6) | 24 (23.1) |
| *Total using a pharmaceutical intervention* | **164 (55.2)** | **38 (36.5)** |
| *Medical cannabis* | 6 (2) | 0 |
|  |  |  |
| *Hospital intervention* |  |  |
| *1 ≤ specialist consultants/ surgeons* | 33 (11.1) | 5 (4.8) |
| *1 ≤ previous surgeries* | 34 (11.4) | 9 (8.7) |
| *Awaiting surgery* | 15 (5.1) | 1 (1) |
| *Total supported through 1 ≤ hospital/surgical intervention* | **60 (20.2)** | **12 (11.5)** |
|  |  |  |
| *Not receiving medical intervention* | 59 (19.9) | 32 (30.8) |
| *No response* | 2 (0.7) | 0 |

*^1^ Sports massage therapy, physiotherapy, osteopath, hydrotherapy, dry needling, chiropractor/ EDS specialised Pilates/ reflexology, therapy, acupuncture, movement therapy, hand therapy cranial sacral therapy, pelvic physio, exercise physiologist, podiatry.*

*^2^ Psychologist, psychological therapy, mental wellbeing service, meditation, counselling*

*^3^ Splints, wrist splint, knee support, ankle brace, finger/thumb splint, osteopathic adjustments, orthotics, Lycra suit, compression stockings*

*Table 4. The results of the regression analyses*

| Criterion Variable | Overall Regression Model | Adjusted 𝑅_2_ | Correlation Coefficient | Significant Predictors | Non-significant Predictors |
| --- | --- | --- | --- | --- | --- |
| *Generalized Anxiety* | 𝐹(4, 395) = 19.72, 𝑝 < .001 | 0.158 | 0.41 | Loneliness (𝑡 = 3.79, 𝑝 < .001)  PSS-Friends (𝑡 = 3.84, 𝑝 < .001) | Dog Guardianship (𝑡 = 0.009, 𝑝 = .993)  Family Support (𝑡 = 1.74, 𝑝 = .083) |
| *Pain (CPAQ-8)* | 𝐹(4, 383) = 11.10, p < .001 | 0.094 | 0.10 | Loneliness (𝑡 = 3.50, 𝑝 < .001)  PSS-Friends (𝑡 = 2.58, 𝑝 = .010) | Dog Guardianship (𝑡 = 0.13, 𝑝 = .899)  Family Support (𝑡 = 0.57, 𝑝 = .566) |
| *Pain (RAND)* | 𝐹(4, 395) = 5.94, 𝑝 < .001 | 0.047 | 0.06 | Loneliness (𝑡 = 3.57, 𝑝 < .001) | Dog Guardianship (𝑡 = 0.05, 𝑝 = .961)  Friend Support (𝑡 = 0.804, 𝑝 = .422)  Family Support (𝑡 = 0.043, 𝑝 = .966) |
| *Physical functioning* | *F*(4, 395) = 14.64, *p* < .001 | 0.120 | .36 | Loneliness (*t* = 5.24, p < .001)  PSS-Friends (*t* = 2.28, *p* = .023) | Dog guardianship (*t* = 0.22; *p* =.830)  Family Support (*t* = 1.493; *p* = .136) |
| *Social functioning* | *F*(4, 399) = 28.01, *p* < .001 | .213 | .470 | Loneliness (*t* = 4.49, *p* < .001)  Friend Support (*t* = 3.99, *p* < .001)  Family Support (*t* = 3.07, *p* = .002) | Dog Guardianship (*t* = .05; *p* = .960) |
| *Role Limitations (Emotional Health)* | 𝐹(4, 393) = 10.96, *p* < .001 | 0.091 | 0.32 | Loneliness (𝑡 = 3.07, 𝑝 = .002)  Dog Guardianship (𝑡 = 2.22, 𝑝 = .027)  Friend Support (𝑡 = 2.73, 𝑝 = .007) | Family Support (𝑡 = 0.07, 𝑝 = .947) |
| *Energy/Fatigue* | 𝐹(4, 395) = 5.41, 𝑝 < .001 | 0.052 | 0.23 | Loneliness (𝑡 = 3.27, 𝑝 < .001) | Dog Guardianship (𝑡 = 0.33, 𝑝 = .742)  Friend Support (𝑡 = 0.25, 𝑝 = .805)  Family Support (𝑡 = 1.17, 𝑝 = .243) |
| *General Health* | 𝐹(4, 395) = 7.48, 𝑝 < .001 | 0.061 | 0.07 | Loneliness (𝑡 = 3.20, 𝑝 < .001)  Friend Support (𝑡 = 2.00, 𝑝 = .046) | Dog Guardianship (𝑡 =1.30, 𝑝 = .196)  Family Support (𝑡 = 0.32, 𝑝 = .751) |
| *Health Change* | 𝐹(4, 395) = 2.52, 𝑝 = .041 | 0.015 | 0.03 | None | Loneliness (𝑡 =1.52, 𝑝 = .129)  Friend Support (𝑡 = 0.287, 𝑝 = .774)  Dog Guardianship (𝑡 = 1.84, 𝑝 = .067)  Family Support (𝑡 = 0.782, 𝑝 = .435) |
| *Wellbeing* | 𝐹(4, 395)=46.91, 𝑝 < .001 | 0.315 | 0.32 | Loneliness (𝑡 = 5.70, 𝑝 < .001)  Friend Support (𝑡 = 5.58, 𝑝 < .001)  Family Support (𝑡 = 3.15, 𝑝 < .001) | Dog Guardianship (𝑡 = 0.06, 𝑝 = .954) |
| *Health-Related Quality of Life* | 𝐹(4, 395) = 3.84, *p* = .005 | 0.028 | 0.04 | None | Loneliness (𝑡 = 1.83, 𝑝 = .067)  Friend Support (𝑡 = 1.07, 𝑝 = .284)  Family Support (𝑡 = 1.41, 𝑝 = .158)  Dog Guardianship (𝑡 = 0.56, 𝑝 = .574) |

*Table 5. The Unstandardised regression coefficients, standardised Beta coefficients, zero-order correlations and semi-partial correlations for each regression*

| Predictor | Generalized Anxiety | Pain (CPAQ-8) | Pain (RAND) | Role Limitations (Emotional Health) | Energy/  Fatigue | General Health | Health Change | Wellbeing | Quality of life | Physical functioning | Social functioning |
| --- | --- | --- | --- | --- | --- | --- | --- | --- | --- | --- | --- |
| Unstandardized Regression Coefficients (B) |  |  |  |  |  |  |  |  |  |  |  |
| *Loneliness* | 0.680 | -0.979 | -1.594 | -3.475 | -1.535 | -1.594 | -1.244 | -1.345 | *-0.778* | *-3.685* | *-2.572* |
| *Dog Guardianship* | 0.006 | -0.127 | -2.342 | 9.111 | -0.563 | -2.342 | 5.456 | 0.050 | *-0.866* | *0.550* | *-0.104* |
| *Family Support* | -0.082 | 0.042 | -0.041 | 0.020 | 0.144 | -0.041 | 0.168 | 0.218 | *0.158* | *-0.276* | *0.462* |
| *Friend Support* | -0.230 | 0.238 | 0.332 | 1.030 | 0.039 | 0.332 | 0.078 | 0.439 | *0.152* | *0.535* | *0.763* |
| Standardized Beta Coefficients (Beta) |  |  |  |  |  |  |  |  |  |  |  |
| *Loneliness* | 0.209 | -0.203 | -0.187 | -0.177 | -0.192 | -0.187 | -0.091 | -2.83 | *-0.109* | *-0.296* | *-0.239* |
| *Dog Guardianship* |  |  |  |  |  |  |  |  |  |  |  |
| *Family Support* | -0.086 | 0.030 | -0.017 | 0.003 | 0.062 | -0.017 | 0.042 | 0.157 | *0.075* | *-0.076* | *0.147* |
| *Friend Support* | -0.212 | 0.150 | 0.117 | 0.157 | 0.015 | 0.117 | 0.017 | 0.277 | *0.064* | *0.129* | *0.213* |
| Zero-order Correlations (r) |  |  |  |  |  |  |  |  |  |  |  |
| *Loneliness* | 0.187 | -0.176 | -0.159 | -0.153 | -0.162 | -0.159 | -0.076 | -0.275 | *-0.092* | *-0.255* | *-0.220* |
| *Dog Guardianship* | 0.000 | 0.006 | 0.065 | 0.111 | 0.017 | 0.065 | 0.092 | 0.003 | *0.028* | *.011* | *0.003* |
| *Family Support* | -0.087 | 0.029 | -0.016 | 0.003 | 0.059 | -0.016 | 0.039 | 0.174 | *0.071* | *-0.075* | *0.153* |
| *Friend Support* | -0.190 | 0.031 | 0.100 | 0.136 | 0.012 | 0.100 | 0.014 | 0.270 | *0.054* | *0.114* | *0.197* |
| Semi-partial Correlations (part r) |  |  |  |  |  |  |  |  |  |  |  |
| *Loneliness* | 0.174 | -0.169 | -0.155 | -0.147 | -0.160 | -0.155 | -0.076 | -0.236 | *-0.091* | *-0.246* | *-0.199* |
| *Dog Guardianship* | 0.000 | 0.006 | 0.063 | 0.106 | 0.016 | 0.063 | 0.091 | 0.002 | *0.028* | *0.010* | *0.002* |
| *Family Support* | -0.080 | 0.028 | -0.015 | 0.003 | 0.057 | -0.015 | 0.039 | 0.146 | *0.070* | *-0.070* | *0.136* |
| *Friend Support* | -0.177 | 0.125 | 0.097 | 0.131 | 0.012 | 0.097 | 0.014 | 0.231 | *0.053* | *0.107* | *0.177* |

Below is the r script for the robust regression analyses, which was adapted for each criterion variable.

\# Load the necessary packages

library(MASS) # For robust regressions

library(foreign) # For reading data files in different formats

\# Set working directory

setwd("NAME OF WORKING DIRECTORY")

\# Set the file path to your data file

file_path <- "NAME OF FILEPATH"

\# Says that missing data is represented by 9999

data <- read.csv(file_path, na.strings = "9999")

\# Rename the column to remove the BOM character

colnames(data)[colnames(data) == "?..Pet"] <- "Pet"

\# Remove rows with missing values in any of the relevant columns

data_clean <- na.omit(data[, c("PHQTotal", "PSSFRTotal", "UCLATotal", "PSSFATotal", "Pet")])

nrow(data_clean)	# Check the number of rows after removing missing values

\# Perform robust OLS regression on cleaned data

ols <- lm(PHQTotal ~ PSSFRTotal + UCLATotal + PSSFATotal + Pet, data = data_clean)

summary(ols)

\## Plotting diagnostics

\# Plot residuals vs Fitted scatterplot and Q-Q plots to check homoscedasticity and normality of residuals

opar <- par(mfrow = c(2,2), oma = c(0, 0, 1.1, 0))

plot(ols, las = 1)

par(opar)

\# Identify problematic data (input the numbers on the graph) that have either high leverage or large residuals

\# data[c(161, 134, 191), 1:2] #Example

\# Display p values with relatively large Cook's D (convention cut off is 4/sample size) - Cook's (column d1) greater than .5 is often seen as problematic

\# Calculate Cook's distance

d1 <- cooks.distance(ols)

\# Calculate standardized residuals manually

residuals <- residuals(ols)

fitted <- fitted(ols)

std_residuals <- residuals / sqrt(1 - d1)

\# Combine data with Cook's distance and standardized residuals

a <- cbind(data_clean, d1, std_residuals)

\# Subset data based on Cook's distance

subset_data <- a[d1 > 4/nrow(data_clean), ]

\# Create a new variable called absr1, which is the absolute value of the residuals and print the 10 p's with the highest absolute residual values

rabs <- abs(std_residuals)

a <- cbind(data_clean, d1, std_residuals, rabs)

asorted <- a[order(-rabs), ]

asorted[1:10, ]

\# Robust regression of iterated re-weighted least squares (IRLS) using Huber weights, which reduces the influence of outliers

summary(rr.huber <- rlm(PHQTotal ~ PSSFRTotal + UCLATotal + PSSFATotal + Pet, data = data_clean))

\# Create data frame with predictors, residuals, and weights for Huber Weights

hweights <- data.frame(PSSFRTotal = data_clean$PSSFRTotal, UCLATotal = data_clean$UCLATotal, PSSFATotal = data_clean$PSSFATotal, Pet = data_clean$Pet, resid = rr.huber$resid, weight = rr.huber$w)

\# Sort the data frame by weights

hweights2 <- hweights[order(rr.huber$w), ]

\# Display the first 15 rows of hweights2

head(hweights2, 15)

\# Same model with Bisquare Weighting function

rr.bisquare <- rlm(PHQTotal ~ PSSFRTotal + UCLATotal + PSSFATotal + Pet, data = data_clean, psi = psi.bisquare)

summary(rr.bisquare)

\# Create the biweights data frame with predictors, residuals, and weights

biweights <- data.frame(PSSFRTotal = data_clean$PSSFRTotal, UCLATotal = data_clean$UCLATotal, PSSFATotal = data_clean$PSSFATotal, Pet = data_clean$Pet, resid = rr.bisquare$resid, weight = rr.bisquare$w)

\# Sort the data frame by weights

biweights2 <- biweights[order(rr.bisquare$w), ]

\# Perform robust regression using the rlm function

robust_model <- rlm(PHQTotal ~ PSSFRTotal + UCLATotal + PSSFATotal + Pet, data = data_clean)

\# Print the summary of the robust regression model

summary(robust_model)
